# Dual Emission with Efficient Phosphorescence Promoted by Intermolecular Halogen Interactions in Luminescent Tetranuclear Zinc(II) Clusters

**DOI:** 10.1021/acs.inorgchem.4c02058

**Published:** 2024-08-09

**Authors:** Fumiya Kobayashi, Yuta Takatsu, Daisuke Saito, Masaki Yoshida, Masako Kato, Makoto Tadokoro

**Affiliations:** † Department of Chemistry, Faculty of Science, Tokyo University of Science, 1-3 Kagurazaka, Shinjuku-ku, Tokyo 162-8601, Japan; ‡ Department of Applied Chemistry for Environment, School of Biological and Environmental Sciences, Kwansei Gakuin University, 1, Gakuen Uegahara, Sanda, Hyogo 669-1330, Japan

## Abstract

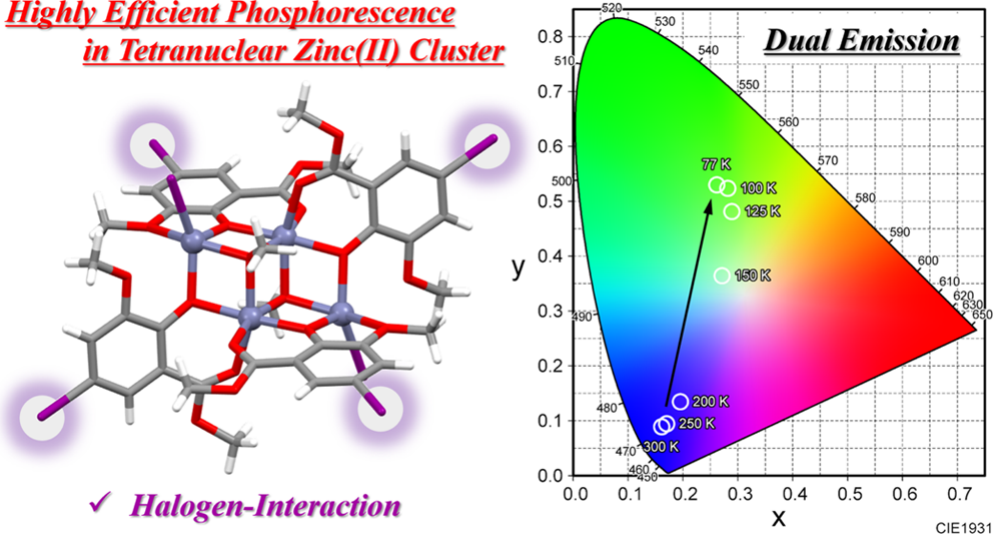

The development of Zn-based phosphorescent materials, associated with a ligand-centered (LC) transition, is extremely limited. Herein, we demonstrated dual emissions including fluorescence and phosphorescence in luminescent tetranuclear Zn(II) clusters [Zn_4_L^I^_4_(μ_3_-OMe)_2_X_2_] (**HL^I^** = methyl-5-iode-3-methoxysalicylate; X = I, Br, Cl), incorporating iodine-substituted ligands. Single-crystal X-ray structural analyses and variable-temperature emission spectra studies revealed the presence of iodine substitutions, and intermolecular halogen interactions produced the internal/external heavy-atom effects and yielded strong green phosphorescence with a long emission lifetime (λ_max_ = 510–522 nm, Φ_em_ = 0.28–0.47, τ_av_ = 0.78–0.95 ms, at 77 K). This work provided a new example that the introduction of halogen interactions is an advantageous approach for inducing phosphorescence in fluorescent metal complexes.

## Abstract 2

An example of dual emissions and temperature-dependent emission color modulation promoted by intermolecular halogen interactions, including the expression of efficient phosphorescence, has been demonstrated in tetranuclear zinc(II) clusters. In this system, the substituted iodine atom is the key for the corresponding luminescence properties.

## Introduction

Luminescent Zn(II) complexes have attracted attention as a functional material in organic light-emitting devices and molecular probes; owing to their low cost and low toxicity, these complexes are an alternative to rare metal ions with strong phosphorescence, such as Ir^III^, Pt^II^, Ru^II^, and Au^I^.^1−9^ However, the intersystem crossing (ISC) promoted by spin–orbit coupling (SOC) is intrinsically weak in Zn(II) complexes, as their emission origin is mainly associated with a ligand-centered (LC) transition, resulting in fluorescent emission.^6−9^ Thus, achieving a high phosphorescence quantum yield (QY), which is industrially important for Zn(II) complexes, remains challenging. In the area of pure organic molecules, one direct and efficient way to facilitate ISC is the introduction of heavy atoms (e.g., halogens).^10−12^ The external heavy-atom effect (EHE) through orbital interactions between the heavy atoms and the luminophore increases both the ISC rate and the phosphorescence decay time of the excited-state luminophore.^13−16^ Kim et al. have reported purely organic phosphorescence molecules promoted by crystal engineering, through halogen bonding and the resulting EHE.^17^ In the recent decade, there has been a notable increase in reports concerning phosphorescence materials that use halogen bonding, indicating their significance and desirability.^18−23^ Among these, the development of a highly luminescent material that exhibits dual emission from the singlet excited state (fluorescence) and triplet excited state (phosphorescence)^24−29^ is significant owing to its contribution to time-resolved imaging technology, which has application in various fields such as in anticounterfeiting systems.^30−33^

**Scheme 1:**
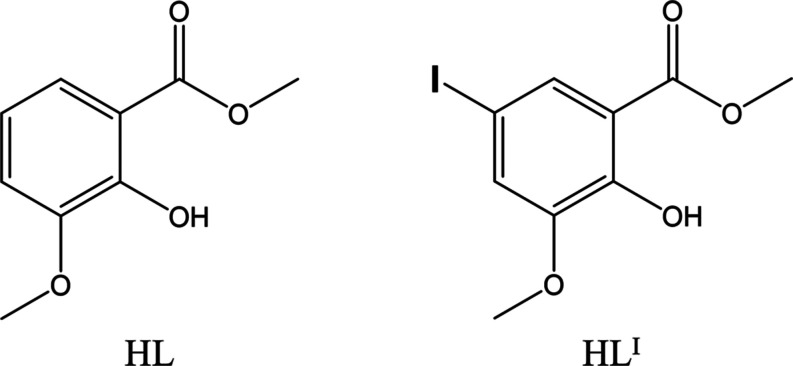
Molecular Structure of **HL** (3-Methoxysalicylic Acid Methyl Ester) and **HL^I^** (5-Iode-3-methoxysalicylic Acid Methyl Ester)

Although halogen bonding is commonly used to enhance the phosphorescence of organic molecules, there have been very few reports on the application of EHE through halogen bonding in metal complex systems. The further modification of fluorescent metal complexes using halogen bonding is an attractive avenue to develop pioneering multifunctional phosphorescent metal complexes. In our previous study, we demonstrated that a heptanuclear Zn(II) cluster [Zn_7_L_6_(μ_3_-OMe)_2_(μ_3_-OH)_4_]I_2_ (**HL** = methyl-3-methoxysalicylate), incorporating iodide counteranions, exhibited strong phosphorescence with an exceptionally long emission lifetime, which can be attributed to the EHE.^34^ This study offered a unique case of EHE-induced phosphorescence in metal complex systems, where halogen interactions acted as the trigger for the corresponding phosphorescence. This mechanism provides an efficient means of introducing SOC into luminescent metal complexes, particularly those associated with LC transitions. Consequently, extending the application of this system to similar types of multinuclear Zn(II) clusters is promising for the further development of multifunctional luminescent materials. Herein, we report novel tetranuclear Zn(II) clusters [Zn_4_L_4_^I^(μ_3_-OMe)_2_X_2_] (**HL^I^** = methyl-5-iode-3-methoxysalicylate; X = I, Br, Cl), where the ligand **HL^I^** is iodine-substituted (Scheme 1). These Zn(II) clusters exhibit highly efficient phosphorescence and temperature-dependent emission color modulation, which are characteristics not observed in substitution-free Zn(II) clusters [Zn_4_L_4_(μ_3_-OMe)_2_X_2_] (X = NCS, Cl, Br).^34^

## Experimental Section

### Synthesis

All reagents and solvents were obtained from Tokyo Kasei Co. and Wako Pure Chemical Industries and were of reagent grade; they were used without further purification. All reactions were carried out under an ambient atmosphere.

### Preparation of Methyl-5-iode-3-methoxysalicylate (**HL^I^**)

**HL^I^** was prepared according to the method reported previously.^35^ The single crystal suitable for single X-ray structural analysis was obtained by recrystallization from ethyl acetate/hexane (4:1) as the colorless crystals. ^1^H NMR (400 MHz, DMSO-*d*_6_): 3.80 (s, 3H), 3.85 (s, 3H), 7.40 (d, 1H), 7.58 (d, 1H), 10.41 (s, 1H) (Figure S1). IR (KBr/cm^–1^): 3134, 3086, 2947, 2723, 1676, 1572, 1473, 1439, 1392, 1346, 1290, 1254, 1233, 1198, 11,167, 1059, 885, 862, 785, 725, 690, 629, 571.

### Preparation of Tetranuclear Zinc(II) Complexes

Tetranuclear Zn(II) clusters were synthesized according to the method we described previously (with minor modifications).^36^

### [Zn_4_L_4_^I^(μ_3_-OMe)_2_I_2_] (**1**)

Triethylamine (0.202 g, 2.00 mmol) in methanol (10 mL) was added with stirring to methanol (20 mL) containing **HL^I^** (0.31 g, 1.00 mmol) and ZnI_2_ (0.31 g, 1.00 mmol). The reaction mixture was stirred for 30 min at room temperature under air. The white microcrystals were participated and collected by suction filtration, washed with a small amount of methanol, and dried in air. Yield 78%. The single crystal suitable for the single X-ray structural analysis was obtained by allowing the mixed solution to stand for a few days to yield **1** as colorless block crystals. Anal. Calc. for **1** (C_38_H_38_I_6_O_18_Zn_4_): C, 25.28; H, 2.12; I, 42.17%. Found: C, 24.95; H, 2.34; I, 41.32%. IR (KBr/cm^–1^): 3087, 2956, 2819, 1664, 1647, 1583, 1552, 1464, 1441, 1348, 1323, 1228, 1186, 1105, 1061, 976, 895, 849, 808, 793, 704, 602, 567, 553.

### [Zn_4_L_4_^I^(μ_3_-OMe)_2_Br_2_] (**2**)

Complex **2** was prepared by a procedure similar to that employed for **1**, except that ZnBr_2_ was used instead of ZnI_2_. **2** was obtained as colorless crystals which were collected by suction filtration, washed with a small amount of methanol, and dried in air. Yield 47%. Anal. Calc. for **2**·H_2_O (C_38_H_38_I_4_Br_2_O_18_Zn_4_): C, 26.12; H, 2.42; Br, 9.14%. Found: C, 25.83; H, 2.35; Br, 9.39%. IR (KBr/cm^–1^): 3087, 2952, 2827, 1662, 1645, 1583, 1552, 1464, 1442, 1348, 1323, 1228, 1188, 1107, 1063, 974, 983, 850, 808, 793, 704, 667, 592, 566, 553.

### [Zn_4_L_4_^I^(μ_3_-OMe)_2_Cl_2_] (**3**)

Complex **3** was prepared by a procedure similar to that employed for **1**, except that ZnCl_2_ was used instead of ZnI_2_. **3** was obtained as colorless crystals, which were collected by suction filtration, washed with a small amount of methanol, and dried in air. Yield 54%. Anal. Calc. for **3** (C_38_H_38_I_4_Cl_2_O_18_Zn_4_): C, 28.13; H, 2.36; I, 31.28; Cl, 4.37%. Found: C, 28.03; H, 2.14; I, 31.28; Cl, 4.84%. IR (KBr/cm^–1^): 3086, 2945, 2843, 1674, 1606, 1572, 1473, 1439, 1394, 1346, 1315, 1288, 1254, 1232, 1198, 1167, 1061, 978, 885, 862, 845, 785, 725, 692, 629, 571.

### Physical Measurements

^1^H NMR spectrum for **HL^I^** was measured with a JEOL JNM-ECZS instrument. Elemental analyses (C, H, N, Cl, Br, and I) were performed on a J-Science Lab JM10 CHN analyzer. Infrared (IR) spectra measurements were performed on a HORIBA FT-730 instrument equipped with the KBr pellet method. Fast atom bombardment (FAB) mass spectra for **1** were measured with a JEOL JMS-AX505HA instrument with 3-nitrobenzyl alcohol (NBA) matrix.

### Single-Crystal and Powder X-ray Diffraction

The single-crystal X-ray diffraction data for **1**–**3** and **HL^I^** were recorded on a Bruker D8 QUEST diffractometer employing graphite monochromated Mo Kα radiation generated from a sealed tube (λ = 0.7107 Å). Data integration and reduction were undertaken with APEX3. Using Olex2 software, the structure was solved with the SHELXT structure solution program using Intrinsic-Phasing Methods and refined with the SHELXL refinement package using least squares minimization. Hydrogen atoms were included in idealized positions and refined using a riding model. Powder X-ray diffraction data (PXRD) for **1**–**3** were collected on a Rigaku MiniFlex II (40 kV/15 mA) X-ray diffractometer using Cu Kα radiation (λ = 1.5406 Å) in the 2θ range of 5–30° with a step width of 3.0°.

### Luminescence Property Measurements

Emission spectra at 298 and 77 K (Figures 2a,b and S5–S7) were measured using a JASCO FP-6600 spectrofluorometer. Emission quantum yields were recorded using a Hamamatsu Photonics C9920-02 absolute photoluminescence quantum yield measurement system equipped with an integrating sphere apparatus and 150 W CW xenon light source. The accuracy of the instrument was confirmed by the measurement of quantum yield of anthracene in ethanol solution (Φ = 0.27).^37,38^ Emission lifetimes were recorded by using a Hamamatsu Photonics C4780 system equipped with a streak camera (Hamamatsu Photonics C4334) as a photodetector and a nitrogen laser (Usho KEN-X) for the 337 nm excitation. The emission decays of complexes were analyzed using two exponentials, i.e., *I* = *A*_1_ exp(−*t*/τ_1_) + *A*_2_ exp(−*t*/τ_2_), where τ_1_ and τ_2_ denote the lifetimes, and *A*_1_ and *A*_2_ are the pre-exponential factors. Therefore, for the determination of radiative and nonradiative rate constants, the averaged emission lifetimes (τ_av_) were estimated using the following equation:^39^
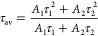
Variable-temperature emission spectra in the solid state and MeOH solution at 300–77 K (Figures 2c,d, and 5) were measured using a SHIMADZU RF-6000 spectrofluorometer equipped with a Unisoku Cryostat (CoolSpek USP-203) thermostated cryostat cell holder.

## Results and Discussion

### Crystal Structures

**Scheme 2:**
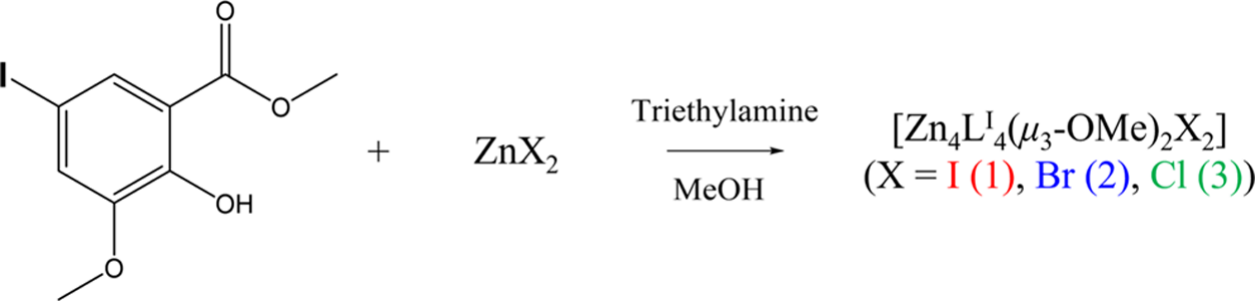
Synthetic Scheme of **1**–**3**

**HL^I^** was prepared according to the method reported previously (Figure S1).^35^ The three tetranuclear Zn(II) clusters [Zn_4_L_4_^I^(μ_3_-OMe)_2_X_2_] (X = I (**1**), Br (**2**), Cl (**3**)) were synthesized using our previously reported methods with minor modifications.^34,36^ Colorless crystals of **1**–**3** were obtained by allowing a mixed solution of the required Zn(II) salt, **HL^I^**, and triethylamine in methanol to stand at room temperature for a few days (Scheme 2). The obtained compounds were characterized by elemental analysis and single-crystal and powder X-ray diffraction (XRD) measurements.

**Figure 1:**
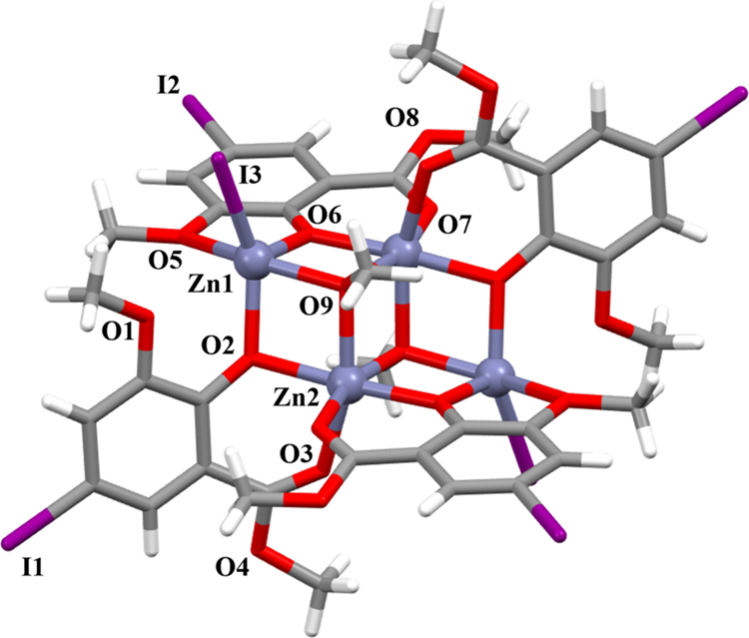
Crystal structure of tetranuclear Zn(II) cluster [Zn_4_L_4_^I^(μ_3_-OMe)_2_I_2_] (**1**) at 173 K. Color code: gray, Zn; red, O; light gray, C; purple, I; white, H.

Single-crystal X-ray structural analyses for **1**–**3** were carried out at 173 K. Individual structures of the tetranuclear Zn(II) clusters **1**–**3** are shown in Figures 1 and S2a, with the crystallographic data presented in Table S1. Each compound features a “defective” double-cubane core [Zn_4_O_6_], where four Zn(II) ions are bridged by μ_2_-O atoms and μ_3_-methoxo groups from the deprotonated **L^I^–^^** ligands (Figure S3b). One of the ligands **L^I^–^^** is disordered. In **1**, two distinct octahedral Zn(II) coordination spheres are present (Figure S3a). One sphere is formed by six oxygen atoms: two from bridging methoxo groups and four from bridging phenoxo group and carbonyl group on the **L^I^–^^** ligands. The other sphere consists of an anion and four oxygen atoms: one from a bridging methoxo group and three from bridging phenoxo and methoxy groups on the **L^I^–^^** ligands. **2** and **3** have structures identical to **1** (Figures S2 and S4). The tetranuclear structures of **1**–**3** are similar to those reported for Zn(II) clusters, which incorporate **HL** ligand derivatives.^34,40^

### Luminescence Properties

**Figure 2:**
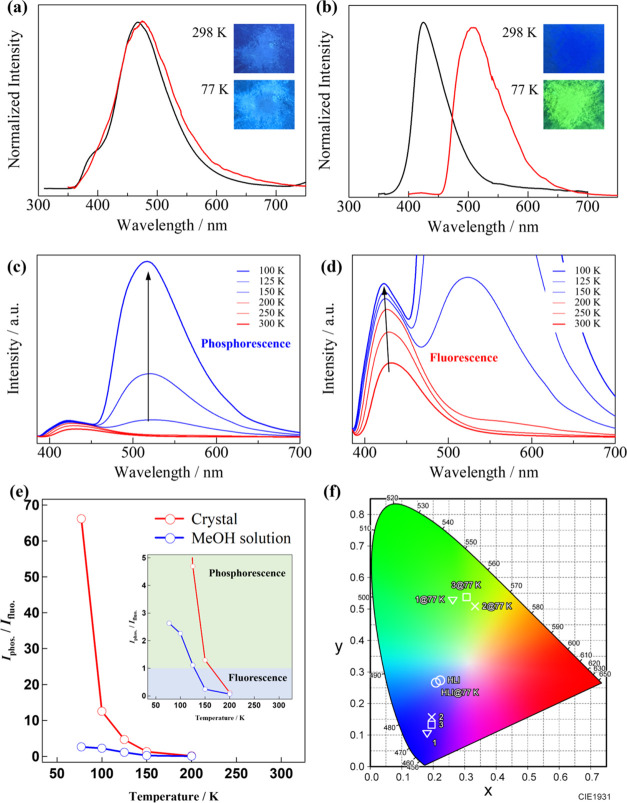
Emission spectra for (a) **HL^I^** and (b) **1** in the solid state at 298 K (black) and 77 K (red) (inset: emission images under UV light (365 nm)). (c, d) Temperature dependence of emission spectra for **1** at 300–100 K. (e) Emission-integrated intensity ratio (*I*_phosphorescence_/*I*_fluorescence_) for **1** at 200–77 K in the solid state (red) and MeOH solution (blue) (inset: enlarged figure). (f) CIE 1931 chromaticity diagrams for emission spectra of **HL^I^** and **1**–**3** in the solid state at 298 and 77 K.

The solid-state excitation and emission spectra of the **HL^I^** ligand at 298 and 77 K are shown in Figures 2a and S5 (Table S2). At 298 K, the crystalline solid sample of **HL^I^** exhibited light blue emission. The emission spectrum exhibited a fluorescence band with a maxima (λ_max_) at 473 nm attributed to the ^1^ππ* transition. The solid-state excitation and emission spectra of compounds **1**–**3** at 298 and 77 K are presented in Figures 2b and S6, S7 (Table S2). At 298 K, crystalline solid samples of **1**–**3** displayed weak blue luminescence. The emission spectra for **1**–**3** featured broad, unstructured emission bands with maxima (λ_max_) at 425, 438, and 425 nm, respectively. While slight variations in the emission maxima for **1**–**3** were noted, the overall emission profiles were similar. The observations described above for **1**–**3** suggest that the emission maxima of **1**–**3** are largely independent of the coordinating anions. Importantly, in the spectra at 77 K, emission maxima of 510–522 nm were observed, which can be attributed to phosphorescence, alongside the emission bands at 425–438 nm. In addition, we evaluated the temperature dependence of the emission behavior at 300–100 K for **1** (Figure 2c,d). Although the emission intensities of the maxima increased slightly, the emission spectra remained almost unchanged below 250 K. Additional emission bands at 510 nm appeared from 200 K, corresponding to the emission-integrated intensity ratio of *I*_phos._/*I*_fluo._ (=*I*_phosphorescence_/*I*_fluorescence_) increased to 1.3, which was almost 1:1, at 150 K (Figure 2e). Although the phosphorescence intensity increased significantly with decreasing temperature, the fluorescence intensity was almost saturated. The emission intensity ratios of *I*_phos._/*I*_fluo._ at 100 and 77 K were 12.5 and 66.2, respectively, indicating that the main contributor to the presented emission in the low-temperature region was phosphorescence. In our previous work for the heptanuclear Zn(II) cluster [Zn_7_L_6_(μ_3_-OMe)_2_(μ_3_-OH)_4_]I_2_,^34^ the emission intensity ratio of *I*_phos._/*I*_fluo._ was 0.66 at 77 K, and no significant emission color change was observed even at 77 K (Figure S8). Therefore, efficient promotion of the formation of the triplet state by internal and/or external heavy-atom effects via intermolecular halogen interactions is expected for **1**–**3**. The emission images and CIE 1931 coordinates for the emission spectra of **HL^I^** and **1**–**3** clearly illustrate the color change of the emission from deep blue to green for **1**–**3**, attributed to the expression of phosphorescence at low temperature (Figure 2b, inset, and Figure 2f), whereas the emission colors of **HL^I^** were almost unchanged even at 77 K (Figure 2a, inset).

Emission QYs (Φ_em_) and emission lifetimes (τ) were measured for **HL^I^** and **1**–**3** (Tables 1 and S3, Figure S9). The emission QYs of **HL^I^** and **1**–**3** were identical (0.02) at 298 K. Due to the low emission QYs, the emission lifetimes at 298 K could not be detected. At 77 K, the emission QYs of **1**, **2**, and **3** clearly increased to 0.47, 0.31, and 0.28, respectively, whereas that of **HL^I^** was almost unchanged (0.03). The emission lifetimes (τ_av_) of **1**, **2**, and **3** were in the millisecond range of 0.78, 0.91, and 0.95 ms, respectively. These values are consistent with the emission band attributed to phosphorescence involving the lowest-excitation triplet state (T_1_ state). **HL^I^** exhibited a QY value at 0.03 that was significantly smaller than that found for the Zn(II) clusters (0.28–0.47). This difference in values is likely due to the ISC generated by the EHE of the intermolecular halogen interactions present in this case, as described below. The radiative decay rate constants (*k*_r_) for **1**–**3** are 5.99 × 10^2^, 3.39 × 10^2^, and 2.95 × 10^2^ s^–1^, respectively. The order of magnitudes of these values may be reflected in the order of the heavy-atom effect for the coordinated halogen anions (I^–^ > Br^–^ > Cl^–^). Although phosphorescence was not clearly observed at room temperature owing to thermal deactivation, the overall results highlight the significant contribution of the triplet excited states of **1**–**3**, which are generated by the presence of iodine substitutions and EHE. The emission origin of the Zn-based phosphorescent molecular materials reported so far is predominantly associated with metal-to-ligand charge transfer (MLCT),^41−44^ halogen-to-ligand charge transfer (XLCT),^45−47^ and intraligand charge transfer (ILCT),^48^ with extremely limited instances of phosphorescence reported to originate from LC transitions.^34,49^ Additionally, due to a scarcity of reports evaluating photophysical parameters, such as rate constants, the investigation of the estimated *k*_r_ values in this study is highly important. Notably, the fact that these *k*_r_ values for **1**–**3** are comparable to those reported for a Zn(II) complex exhibiting phosphorescence arising from Zn–Zn interactions (4.2 × 10^2^ s^–1^, at 77 K)^50^ is noteworthy.

**Table 1:** Photophysical Properties for **HL^I^** and **1**–**3** in the Solid State at 77 K

	**HL^I^**	**1**	**2**	**3**
λ_em_^a^/nm	475	510	522	519
Φ_em_^b^	0.03	0.47	0.31	0.28
τ_em_^c^/ms		0.784	0.914	0.948
*k*_r_^d^/s^–1^		5.99 × 10^2^	3.39 × 10^2^	2.95 × 10^2^
*k*_nr_^e^/s^–1^		6.76 × 10^2^	7.55 × 10^2^	7.59 × 10^2^
*k*_r_/*k*_nr_		0.89	0.45	0.39

a Emission maximum, λ_ex_ = 300 nm.

b Photoluminescence quantum yields, λ_ex_ = 337 nm.

c Emission lifetime.

d Radiative decay rate constants (*k*_r_) were estimated using the equation: Φ_em_/τ_em_.

e Nonradiative decay rate constants (*k*_nr_) were estimated using the equation: *k*_r_(1 – Φ_em_)/Φ_em_.

**Figure 3:**
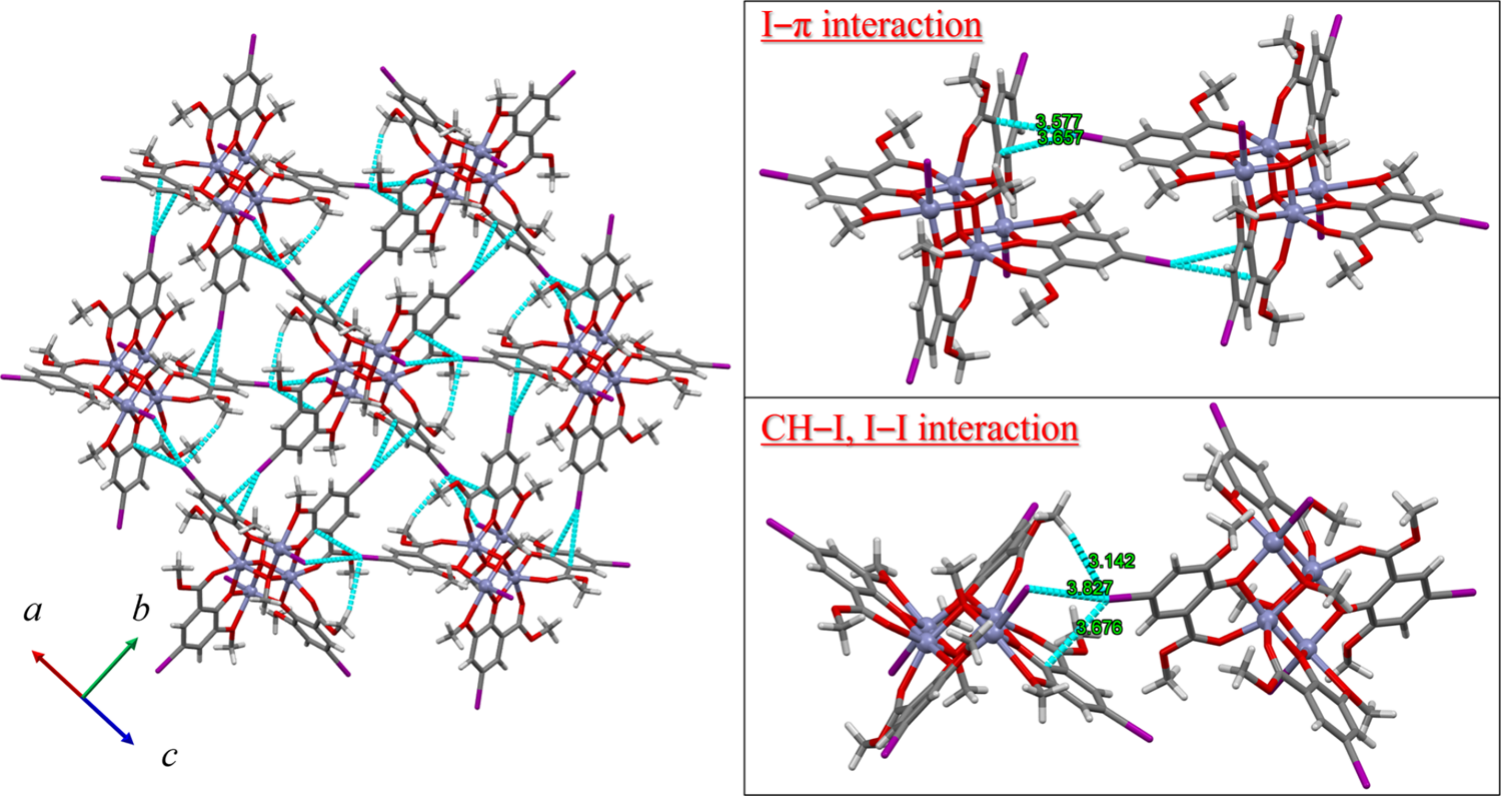
Packing structure of **1** at 173 K. All disordered atoms have been omitted for the sake of clarity. Blue dashed lines represent halogen-related interactions (CH–I, I–I, and I−π interactions).

**Figure 4:**
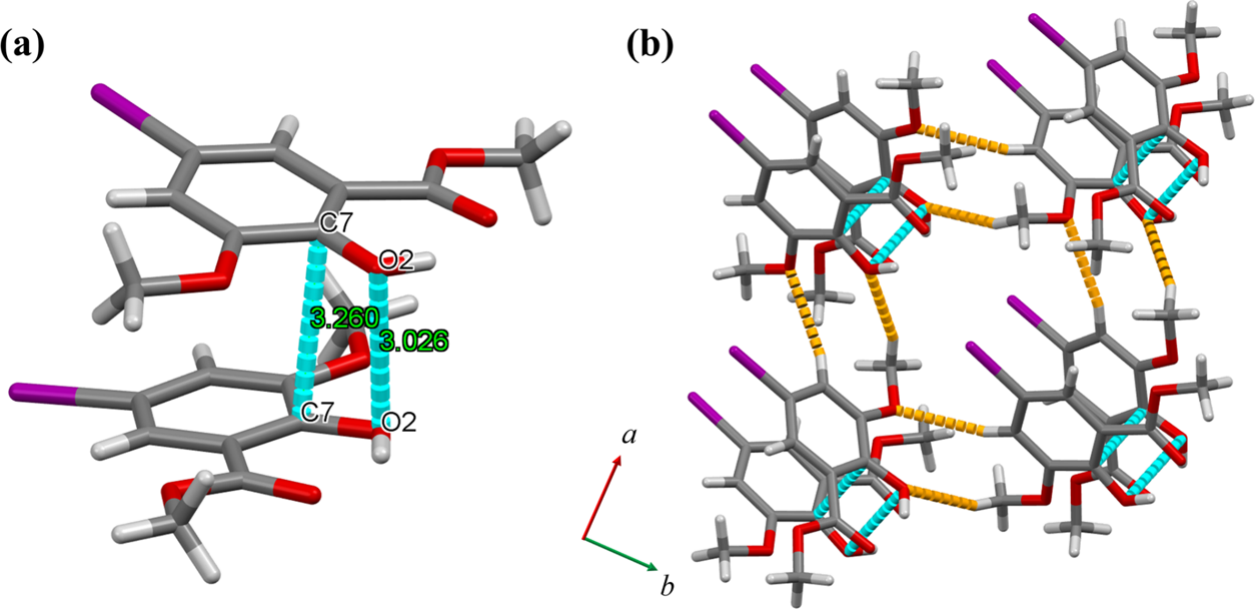
(a) Dimeric structure of **HL^I^** at 173 K. (b) Crystal packing structure of **HL^I^** at 173 K. Blue dashed lines represent π–π interactions, and orange-dashed lines represent CH–O interactions between each dimer.

The observed differences in the photophysical properties of **1**–**3** and **HL^I^** can be attributed to the differences in their crystal structures and the resulting intermolecular interactions. The crystal packing diagrams of compounds **1**–**3** are shown in Figures 3 and S10–S13. For **1**, intermolecular interactions via halogen atoms, such as I−π interaction (I(1)···C(6) = 3.58(1) Å, I(1)···C(7) = 3.657(9) Å), CH–I interactions (C(9)–H(9A)···I(1) = 3.142 Å), and I–I interactions (I(1)···I(3) = 3.827(1) Å), were observed, indicating that all halogen atoms strongly interact with neighboring molecules (Figure 3 and Table S4). Similar intermolecular interactions via halogen atoms were presented in **2** and **3** (Figures S10–S13, Tables S5 and S6). Meanwhile, for the crystal packing structure for **HL^I^** at 173 K, **HL^I^** dimerized by π–π interactions (C(7)···C(7)* = 3.260(4) Å, O(2)···O(2)* = 3.026(3) Å) (Figure 4a and Table S7). Each dimer interacted with neighboring dimers by CH–O interactions (C(1)–H(1A)···O(2) = 2.518 Å, C(3)–H(3)···O(1) = 2.695 Å), forming three-dimensional supramolecular interactions (Figure 4b). Based on the crystal structure analysis, halogen interactions occurred between the **L^I^–^^** ligands of each molecule in **1**–**3**, whereas no halogen-related interactions occurred for **HL^I^**. This is a key factor in the observed phosphorescence differences between **1**–**3** and **HL^I^** in the solid state.

**Figure 5:**
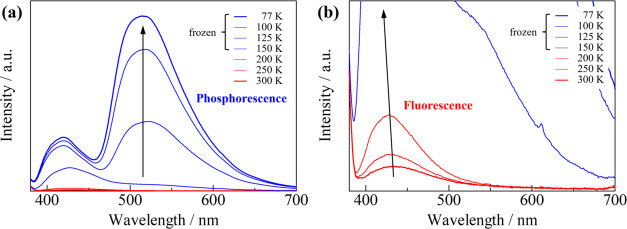
(a) Temperature dependence of emission spectra for **1** at 300–77 K in MeOH (1.0 × 10^–5^ M). (b) Enlarged area in (a).

**Figure 6:**
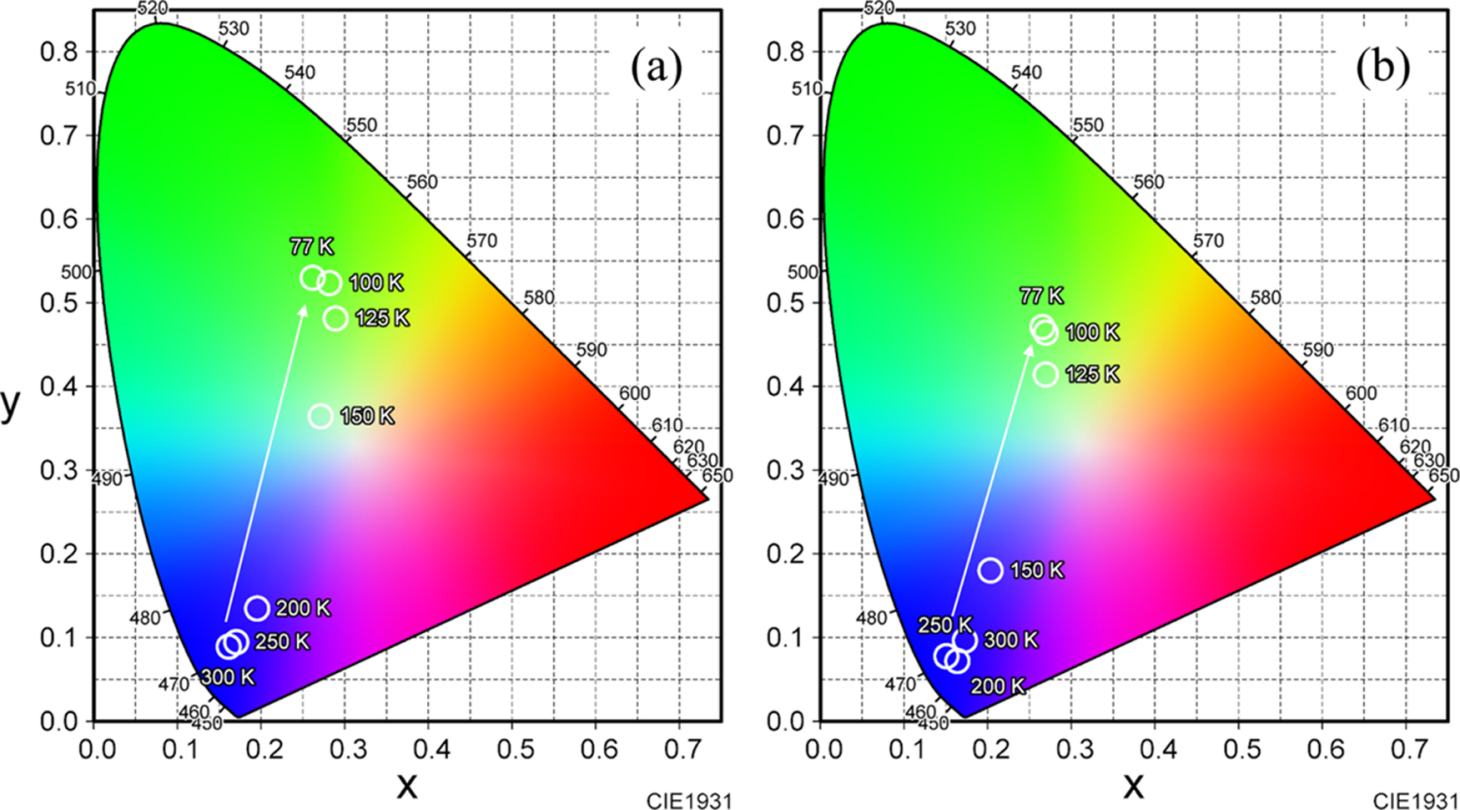
CIE 1931 chromaticity diagrams for the temperature dependence of emission spectra of **1** at 300–77 K in (a) the solid state and (b) MeOH solution.

To evaluate the contribution of the internal heavy-atom effect (IHE) and EHE, we investigated the variable-temperature emission spectrum for **1** in solution (MeOH, 1.0 × 10^–5^ M) (Figure 5). At 300–200 K, **1** exhibited a weak blue emission with an emission maximum at 425 nm. This is because nonradiative transitions reflect the violent molecular vibrations of **1** in solution. At 150 K, the emission intensity at 425 nm increased drastically (approximately 9 times). This can be attributed to the inhibition of molecular vibrations induced by the frozen MeOH solution. However, phosphorescence at 510 nm was not clearly observed at 150 K. Upon further decreasing the temperature, the emission bands remarkably appeared at 510 nm, which can be attributed to the phosphorescence. The emission-integrated intensity ratio of *I*_phos._/*I*_fluo._ at 150 and 77 K was 0.2 and 2.6, respectively (Figure 2e). Compared with that in the solid state, phosphorescence in the solution state decreased, which indicates the key role played by EHE, rather than IHE, induced by intermolecular halogen interactions. To reveal the luminescent species, FAB mass spectrum (MS) analysis was performed for **1** in a MeOH solution. The observed spectrum showed dominant peaks at *m*/*z* = 1267.53, attributable to the heptanuclear species of {[Zn_7_L_6_^I^(μ_3_-OMe)(μ_3_-OH)_5_]I}^2+^, rather than a tetranuclear species. This indicates the possibility that dissolution in MeOH or ionization by MS leads to structural conversion to the heptanuclear species. However, it should be noted that these results do not lead to a precise identification of the correct luminescent species. On the other hand, the results of variable-temperature emission spectra for **HL^I^** in MeOH solution showed clearly different behaviors from those of **1** (Figure S14). At 77 K, **HL^I^** exhibited a structured fluorescence band at 405 nm and a broad phosphorescence band at 510 nm, both with similar intensity. Therefore, the difference in luminescence behavior between the solid state and solution of **1** can still be attributed to EHE due to intermolecular halogen interactions. The CIE 1931 coordinates for the temperature dependence of the emission spectra of **1** clearly demonstrate the emission color differences and modulations from deep blue to green, attributed to the expression of phosphorescence in both the solid state and the solution (Figure 6). The above unique photophysical properties observed in **1**–**3** were not observed in the substitution-free Zn(II) clusters.^34,40^

## Conclusions

In conclusion, we synthesized tetranuclear luminescent Zn(II) clusters [Zn_4_L_4_^I^(μ_3_-OMe)_2_X_2_] (X = I, Br, Cl) and demonstrated that the intermolecular halogen interactions produced the EHE for the expression of strong phosphorescence with long emission lifetime. An improvement over previous studies is noteworthy, as the observed temperature for clear phosphorescence has increased to around 200 K. Further improvement for the development of novel Zn(II) complexes exhibiting room-temperature phosphorescence is currently in progress. Importantly, the formation of halogen interactions by introducing halogen substitutions is an advantageous approach, which can result in highly efficient phosphorescence and functions related to the dual emissions^51,52^ in both organic molecules and fluorescent metal complexes. This result also suggests the possibility of achieving luminescence switching induced by structural rearrangements triggered by other external stimuli, such as mechanical force, and the development of novel optofunctional molecular systems.

## Supporting Information Available

NMR spectrum, crystallographic data, crystal structures, PXRD, excitation and emission spectra, photophysical data, and packing diagrams (PDF)

## References

[ref1] Tung Y.-L.; Lee S.-W.; Chi Y.; Chen L.-S.; Shu C.-F.; Wu F.-I.; Carty A. J.; Chou P.- T.; Peng S.-M.; Lee G.-H. Organic Light-Emitting Diodes based on Charge-Neutral Ru^II^ Phosphorescent Emitters. Adv. Mater. 2005, 17, 1059–1064. 10.1002/adma.200401806.

[ref2] Hsu C.-W.; Lin C.-C.; Chung M.-W.; Chi Y.; Lee G.-H.; Chou P.-T.; Chang C.-H.; Chen P.-Y. Systematic Investigation of the Metal-Structure–Photophysics Relationship of Emissive d^10^-Complexes of Group 11 Elements: The Prospect of Application in Organic Light Emitting Devices. J. Am. Chem. Soc. 2011, 133, 12085–12099. 10.1021/ja2026568. 21711042

[ref3] Lee J.; Chen H.-F.; Batagoda T.; Coburn C.; Djurovich P. I.; Thompson M. E.; Forrest S. R. Deep blue phosphorescent organic light-emitting diodes with very high brightness and efficiency. Nat. Mater. 2016, 15, 92–98. 10.1038/nmat4446. 26480228

[ref4] Lo K. K.-W. Luminescent Rhenium(I) and Iridium(III) Polypyridine Complexes as Biological Probes, Imaging Reagents, and Photocytotoxic Agents. Acc. Chem. Res. 2015, 48, 2985–2995. 10.1021/acs.accounts.5b00211. 26161527

[ref5] Zhang K. Y.; Yu Q.; Wei H.; Liu S.; Zhao Q.; Huang W. Long-Lived Emissive Probes for Time-Resolved Photoluminescence Bioimaging and Biosensing. Chem. Rev. 2018, 118 (4), 1770–1839. 10.1021/acs.chemrev.7b00425. 29393632

[ref6] Wang S. Luminescence and electroluminescence of Al(III), B(III), Be(II) and Zn(II) complexes with nitrogen donors. Coord. Chem. Rev. 2001, 215, 79–98. 10.1016/S0010-8545(00)00403-3.

[ref7] Zheng S.-L.; Chen X.-M. Recent Advances in Luminescent Monomeric, Multinuclear, and polymeric Zn(II) and Cd(II) Coordination Complexes. Aust. J. Chem. 2004, 57, 703–712. 10.1071/CH04008.

[ref8] Barbieri A.; Accorsi G.; Armaroli N. Luminescent complexes beyond the platinum group: the d^10^ avenue. Chem. Commun. 2008, 2185–2193. 10.1039/b716650h. 18463736

[ref9] Wenger O. S. Photoactive Complexes with Earth-Abundant Metals. J. Am. Chem. Soc. 2018, 140, 13522–13533. 10.1021/jacs.8b08822. 30351136

[ref10] Ma X.; Wang J.; Tian H. Assembling-Induced Emission: An Efficient Approach for Amorphous Metal-Free Organic Emitting Materials with Room-Temperature Phosphorescence. Acc. Chem. Res. 2019, 52, 738–748. 10.1021/acs.accounts.8b00620. 30816706

[ref11] Zhao W.; He Z.; Tang B. Z. Room-temperature phosphorescence from organic aggregates. Nat. Rev. Mater. 2020, 5, 869–885. 10.1038/s41578-020-0223-z.

[ref12] Peng Q.; Ma H.; Shuai Z. Theory of Long-Lived Room-Temperature Phosphorescence in Organic Aggregates. Acc. Chem. Res. 2021, 54, 940–949. 10.1021/acs.accounts.0c00556. 33347277

[ref13] Dewar M. J. S.; Patterson D. B.; Simpson W. I. External Heavy-Atom-Induced spin-Orbital Coupling. Spectroscopic Study of Naphthonorbornanes. J. Am. Chem. Soc. 1971, 93, 1032–1034. 10.1021/ja00733a045.

[ref14] Chandra A. K.; Turro N. J.; Lyons A. L. Jr; Stone P. The Intramolecular External Heavy Atom Effect in Bromo-, Benzo-, and Naphthonorbornenes. J. Am. Chem. Soc. 1978, 100, 4964–4968. 10.1021/ja00484a007.

[ref15] Santos M. N. B. External heavy-atom effect on fluorescence kinetics. PhysChemComm 2000, 3, 18–23. 10.1039/b002307h.

[ref16] Zeng Y.; Biczok L.; Linschitz H. External Heavy Atom Induced Phosphorescence Emission of Fullerenes: The Energy of Triplet C_60_. J. Phys. Chem. A 1992, 96, 5237–5239. 10.1021/j100192a014.

[ref17] Bolton O.; Lee K.; Kim H.-J.; Lin K. Y.; Kim J. Activating efficient phosphorescence from purely organic materials by crystal design. Nat. Chem. 2011, 3, 205–210. 10.1038/nchem.984. 21336325

[ref18] Sun X.; Zhang B.; Li X.; Trindle C. O.; Zhang G. External Heavy-Atom Effect via Orbital Interactions Revealed by Single-Crystal X-ray Diffraction. J. Phys. Chem. A 2016, 120, 5791–5797. 10.1021/acs.jpca.6b03867. 27319778

[ref19] Singh M.; Liu K.; Qu S.; Ma H.; Shi H.; An Z.; Huang W. Recent Advances of Cocrystals with Room Temperature Phosphorescence. Adv. Opt. Mater. 2021, 9, 2002197 10.1002/adom.202002197.

[ref20] Yan Z.-A.; Lin X.; Sun S.; Ma X.; Tian H. Activating Room-Temperature Phosphorescence of Organic Luminophores via External Heavy-Atom Effect and Rigidity of Ionic Polymer Matrix. Angew. Chem., Int. Ed. 2021, 60, 19735–19739. 10.1002/anie.202108025. 34240799

[ref21] Xie N.; Yu H.; Wang J.; Li Z.; Wei J.; Wang Y. Phase- and Halogen-Dependent Room-Temperature Phosphorescence Properties of Biphenylnitrile Derivatives. J. Phys. Chem. C 2021, 125, 27489–27496. 10.1021/acs.jpcc.1c09305.

[ref22] Dai W.; Niu X.; Wu X.; Ren Y.; Zhang Y.; Li G.; Su H.; Lei Y.; Xiao J.; Shi J.; Tong B.; Cai Z.; Dong Y. Halogen Bonding: A New Platform for Achieving Multi-Stimuli-Responsive Persistent Phosphorescence. Angew. Chem., Int. Ed. 2022, 61, e202200236 10.1002/anie.202200236. 35102661

[ref23] He Y.; Wang J.; Li Q.; Qu S.; Zhou C.; Yin C.; Ma H.; Shi H.; Meng Z.; An Z. Highly Efficient Room-Temperature Phosphorescence Promoted via Intramolecular-Space Heavy-Atom Effect. Adv. Opt. Mater. 2023, 11, 2201641 10.1002/adom.202201641.

[ref24] Feng C.; Li S.; Xiao X.; Lei Y.; Geng H.; Liao Y.; Liao Q.; Yao J.; Wu Y.; Fu H. Excited-State Modulation for Controlling Fluorescence and Phosphorescence Pathways toward White-Light Emission. Adv. Opt. Mater. 2019, 7, 1900767 10.1002/adom.201900767.

[ref25] Li H.; Li H.; Wang W.; Tao Y.; Wang S.; Yang Q.; Jiang Y.; Zheng C.; Huang W.; Chen R. Stimuli-Responsive Circularly Polarized Organic Ultralong Room Temperature Phosphorescence. Angew. Chem., Int. Ed. 2020, 59, 4756–4762. 10.1002/anie.201915164. 31901181

[ref26] Bi X.; Shi Y.; Peng T.; Yue S.; Wang F.; Zheng L.; Cao Q.-E. Multi-Stimuli Responsive and Multicolor Adjustable Pure Organic Room Temperature Fluorescence-Phosphorescent Dual-Emission Materials. Adv. Funct. Mater. 2021, 31, 2101312 10.1002/adfm.202101312.

[ref27] Wang D.; Xie Y.; Wu X.; Lei Y.; Zhou Y.; Cai Z.; Liu M.; Wu H.; Huang X.; Dong Y. Excitation-Dependent Triplet–Singlet Intensity from Organic Host–Guest Materials: Tunable Color, White-Light Emission, and Room-Temperature Phosphorescence. J. Phys. Chem. Lett. 2021, 12, 1814–1821. 10.1021/acs.jpclett.1c00188. 33577329

[ref28] Roy B.; Maisuls I.; Zhang J.; Niemeyer F. C.; Rizzo F.; Wçlper C.; Daniliuc C. G.; Tang B. Z.; Strassert C. A.; Voskuhl J. Mapping the Regioisomeric Space and Visible Color Range of Purely Organic Dual Emitters with Ultralong Phosphorescence Components: From Violet to Red Towards Pure White Light. Angew. Chem., Int. Ed. 2022, 61, e202111805 10.1002/anie.202111805. PMC9299909 34693600

[ref29] Mao H.; Gao J.; Zhao W.; Wang T.; Shan G.-G.; Geng Y.; Shao K.; Wang X.; Su Z. Boosting ultralong organic phosphorescence performance by synergistic heavy-atom effect and multiple intermolecular interactions in molecular crystal. J. Mater. Chem. C 2022, 10, 6334–6340. 10.1039/D2TC00748G.

[ref30] Kumar P.; Creason T. D.; Fattal H.; Sharma M.; Du M. H.; Saparov B. Composition-Dependent Photoluminescence Properties and Anti-Counterfeiting Applications of A_2_AgX_3_ (A = Rb, Cs; X = Cl, Br, I). Adv. Funct. Mater. 2021, 31, 2104941 10.1002/adfm.202104941.

[ref31] Wei J.-H.; Ou W.-T.; Luo J.-B.; Kuang D.-B. Zero-Dimensional Zn-Based Halides with Ultra-Long Room-Temperature Phosphorescence for Time-Resolved Anti-Counterfeiting. Angew. Chem., Int. Ed. 2022, 61, e202207985 10.1002/anie.202207985. 35703341

[ref32] Li F.; Qian C.; Lu J.; Ma Y.; Zhang K. Y.; Liu S.; Zhao Q. Color-Tunable Dual Persistent Emission Via a Triplet Exciton Reservoir for Temperature Sensing and Anti-Counterfeiting. Adv. Opt. Mater. 2022, 10, 2101773 10.1002/adom.202101773.

[ref33] Miao X.; Cai Z.; Zou H.; Li J.; Zhang S.; Ying L.; Deng W. Achieving halogen bonding enhanced ultra-highly efficient AIE and reversible mechanochromism properties of TPE-based luminogens: position of bromine substituents. J. Mater. Chem. C 2022, 10, 8390–8399. 10.1039/D2TC00712F.

[ref34] Kobayashi F.; Ohtani R.; Teraoka S.; Yoshida M.; Kato M.; Zhang Y.; Lindoy L. F.; Hayami S.; Nakamura M. Phosphorescence at Low Temperature by External Heavy-Atom Effect in Zinc(II) Clusters. Chem. - Eur. J. 2019, 25, 5875–5879. 10.1002/chem.201900343. 30860310

[ref35] Joshua A. V.; Sharma S. K.; Abrams D. N. New Short Synthesis of (5)-2,3-Dimethoxy-N-[(1-ethyl-2-pyrrolidinyl)methyl]-5-iodobenzamide: Dopamine D2 Receptor. Synth. Commun. 2008, 38, 434 10.1080/00397910701771199.

[ref36] Kobayashi F.; Ohtani R.; Teraoka S.; Kosaka W.; Miyasaka H.; Zhang Y.; Lindoy L. F.; Hayami S.; Nakamura M. Syntheses, structures and magnetic properties of tetranuclear cubane-type and heptanuclear wheel-type nickel(II) complexes with 3-methoxysalicylic acid derivatives. Dalton Trans. 2017, 46, 8555–8561. 10.1039/C7DT01757J. 28639637

[ref37] Dawson W. R.; Windsor M. W. Fluorescence Yields of Aromatic Compounds. J. Phys. Chem. A 1968, 72, 3251–3260. 10.1021/j100855a027.

[ref38] Melhuish W. H. QUANTUM EFFICIENCIES OF FLUORESCENCE OF ORGANIC SUBSTANCES: EFFECT OF SOLVENT AND CONCENTRATION OF THE FLUORESCENT SOLUTE. J. Phys. Chem. A 1961, 65, 229–235. 10.1021/j100820a009.

[ref39] Lakowicz J. R. Principles of Fluorescence Spectroscopy, 3rd ed.; Springer: New York, 2006.

[ref40] Kuramitsu T.; Kusumoto S.; Ohmagari H.; Hasegawa M.; Thuery P.; Kim Y.; Hayami S.; Nakamura M. Coordinated Halide and Pseudo Halide-Dependent Structures and Photoluminescence of Defective Double Cubane Zinc(II) Clusters. Eur. J. Inorg. Chem. 2021, 2021, 1160–1164. 10.1002/ejic.202001138.

[ref41] Zhao Y.; Yang X.-G.; Lu X.-M.; Yang C.-D.; Fan N.-N.; Yang Z.-T.; Wang L.-Y.; Ma L.-F. {Zn_6_} Cluster Based Metal–Organic Framework with Enhanced Room-Temperature Phosphorescence and Optoelectronic Performances. Inorg. Chem. 2019, 58, 6215–6221. 10.1021/acs.inorgchem.9b00450. 31002240

[ref42] Yang X.-G.; Zhai Z.-M.; Liu X.-Y.; Li J.-Y.; Li F.-F.; Ma L.-F. Sulfur heteroatom-based MOFs with long-lasting room-temperature phosphorescence and high photoelectric response. Dalton Trans. 2020, 49, 598–602. 10.1039/C9DT04046C. 31850437

[ref43] Yang X.-G.; Zhai Z.-M.; Lu X.-M.; Qin J.-H.; Li F.-F.; Ma L.-F. Hexanuclear Zn(II)-Induced Dense π-Stacking in a Metal–Organic Framework Featuring Long-Lasting Room Temperature Phosphorescence. Inorg. Chem. 2020, 59, 10395–10399. 10.1021/acs.inorgchem.0c01415. 32700527

[ref44] Shu C.; Liu C.; Wu M.; Chen C.; Hong M. Simultaneous fluorescence and phosphorescence in Zn(II)–zwitterionic coordination polymers with tunable colors. J. Mater. Chem. C 2021, 9, 4233–4239. 10.1039/D0TC06109C.

[ref45] Ma Y.-J.; Qi Z.; Xiao G.; Fang X.; Yan D. Metal-Halide Coordination Polymers with Excitation Wavelength and Time-Dependent Ultralong Room-Temperature Phosphorescence. Inorg. Chem. 2022, 61, 16477–16483. 10.1021/acs.inorgchem.2c02750. 36190957

[ref46] Zhou B.; Yan D. Color-tunable persistent luminescence in 1D zinc–organic halide microcrystals for single-component white light and temperature-gating optical waveguides. Chem. Sci. 2022, 13, 7429–7436. 10.1039/D2SC01947G. 35872833 PMC9242015

[ref47] Mu Y.; Cao F.-Y.; Fang X.-Y.; Liu Z.-X.; Wang J.-Q.; Han S.-D.; Pan J.; Wei Q.; Li J.-H.; Wang G.-M. Tunable Full-Color Room Temperature Phosphorescence of Two Single-Component Zinc(II)-Based Coordination Polymers. Adv. Opt. Mater. 2023, 11, 2202402 10.1002/adom.202202402.

[ref48] Lozada I. B.; Braun J. D.; Gareth Williams J. A.; Herbert D. E. Yellow-Emitting, Pseudo-Octahedral Zinc Complexes of Benzannulated N∧N∧O Pincer-Type Ligands. Inorg. Chem. 2022, 61, 17568–17578. 10.1021/acs.inorgchem.2c02585. 36302264

[ref49] Cariati E.; Forni A.; Lucenti E.; Marinotto D.; Previtali A.; Righetto S.; Botta C.; Bold V.; Kravtsov V.; Fonari M. S. Extrinsic Heavy Metal Atom Effect on the Solid-State Room Temperature Phosphorescence of Cyclic Triimidazole. Chem. - Asian J. 2019, 14, 853–858. 10.1002/asia.201801604. 30600907

[ref50] Wada Y.; Maruchi T.; Ishii R.; Sunada Y. Visible Light Responsive Dinuclear Zinc Complex Consisting of Proximally Arranged Two d^10^-Zinc Centers. Angew. Chem., Int. Ed. 2023, 62, e202310571 10.1002/anie.202310571. 37753736

[ref51] Behera S. K.; Park S. Y.; Gierschner J. Dual Emission: Classes, Mechanisms, and Conditions. Angew. Chem., Int. Ed. 2021, 60, 22624–22638. 10.1002/anie.202009789. 32783293

[ref52] Kukhta N. A.; Bryce M. R. Dual emission in purely organic materials for optoelectronic applications. Mater. Horiz. 2021, 8, 33–55. 10.1039/D0MH01316A. 34821289

