# Supplementary material for: Dual Emission with Efficient Phosphorescence Promoted by Intermolecular Halogen Interactions in Luminescent Tetranuclear Zinc(II) Clusters
Source: Inorg Chem. 2024 Aug 9;63(33):15323–30. doi: 10.1021/acs.inorgchem.4c02058 (PMC11337158; doi:10.1021/acs.inorgchem.4c02058)
Supplement: Supplementary file 1 — ic4c02058_si_001.pdf [file ic4c02058_si_001.pdf]

Supporting Information (SI)

Dual Emission with Efficient Phosphorescence Promoted by Intermolecular Halogen-Interactions in Luminescent Tetranuclear Zinc(II) Clusters

Fumiya Kobayashi*,†, Yuta Takatsu,† Daisuke Saito,‡ Masaki Yoshida,‡ Masako Kato,‡ and Makoto Tadokoro*,†

† Department of Chemistry, Faculty of Science, Tokyo University of Science, 1-3 Kagurazaka, Shinjuku-ku, Tokyo 162-8601, Japan

‡ Department of Applied Chemistry for Environment, School of Biological and Environmental Sciences, Kwansei Gakuin University, 1, Gakuen Uegahara, Sanda, Hyogo 669-1330 Japan.

Corresponding author:
F. Kobayashi, fkobayashi@rs.tus.ac.jp
M. Tadokoro, tadokoro@rs.tus.ac.jp

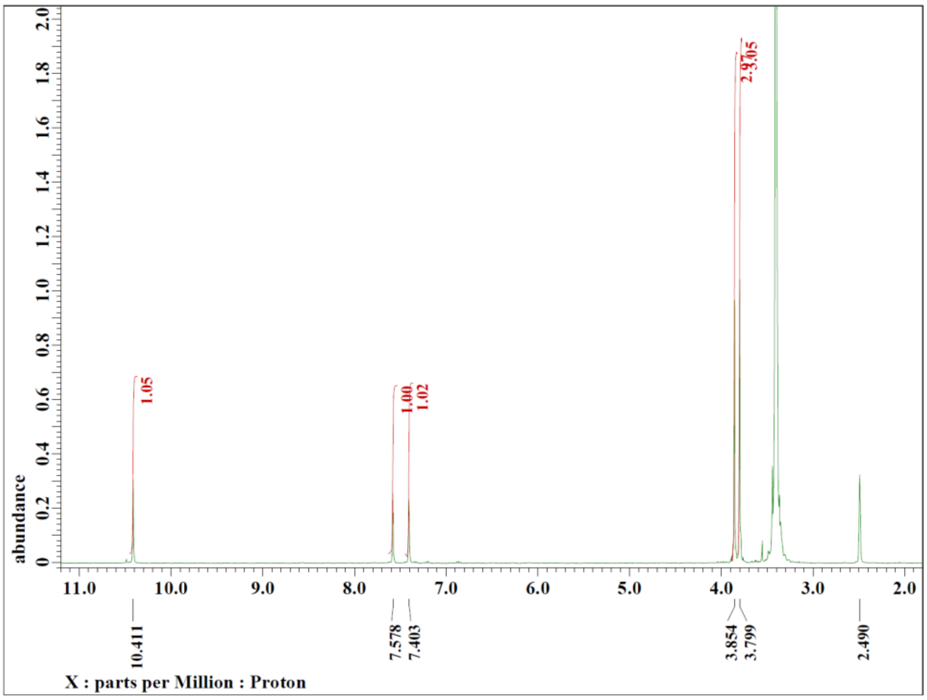

Figure S1. ^{1}H NMR spectrum of **HL^{1}** in DMSO-d_6 (400 MHz, 298 K).

Table S1. Crystallographic data for **1**–**3**.

Compound	**1**	**2**	**3**
Temperature / K	173	173	173
Formula	$C_{38}H_{38}I_6O_{18}Zn_4$	$C_{38}H_{38}I_4Br_2O_{18}Zn_4$	$C_{38}H_{38}I_4Cl_2O_{18}Zn_4$
Crystal system	Monoclinic	Monoclinic	Monoclinic
Space group	$P2_1/n$ (#14)	$P2_1/n$ (#14)	$P2_1/c$ (#14)
a / Å	13.5700(6)	13.1479(11)	12.7459(12)
b / Å	12.8188(7)	12.9260(10)	12.8720(13)
c / Å	16.2155(9)	16.3103(13)	16.5841(16)
α / °	90	90	90
β / °	103.133(2)	100.633(2)	98.647(3)
γ / °	90	90	90
V / Å^3	2746.9(2)	2724.3(4)	2689.9(5)
Z	2	2	2
D_{calc} / g cm^{-3}	2.183	2.086	2.003
μ / mm^{-1}	5.160	5.536	4.220
F(000)	1696	1624	1552
R_1, wR_2 ($I > 2\sigma(I)$)	0.0583, 0.1478	0.0655, 0.1516	0.0845, 0.2062
R_1, wR_2 (for all data)	0.0973, 0.1679	0.0902, 0.1645	0.1384, 0.2352
GOF	1.083	1.074	1.030
Reflections/Parameters	4834/345	4810/407	4755/420
CCDC	2332303	2332304	2332305

(a)

(b)

(c)

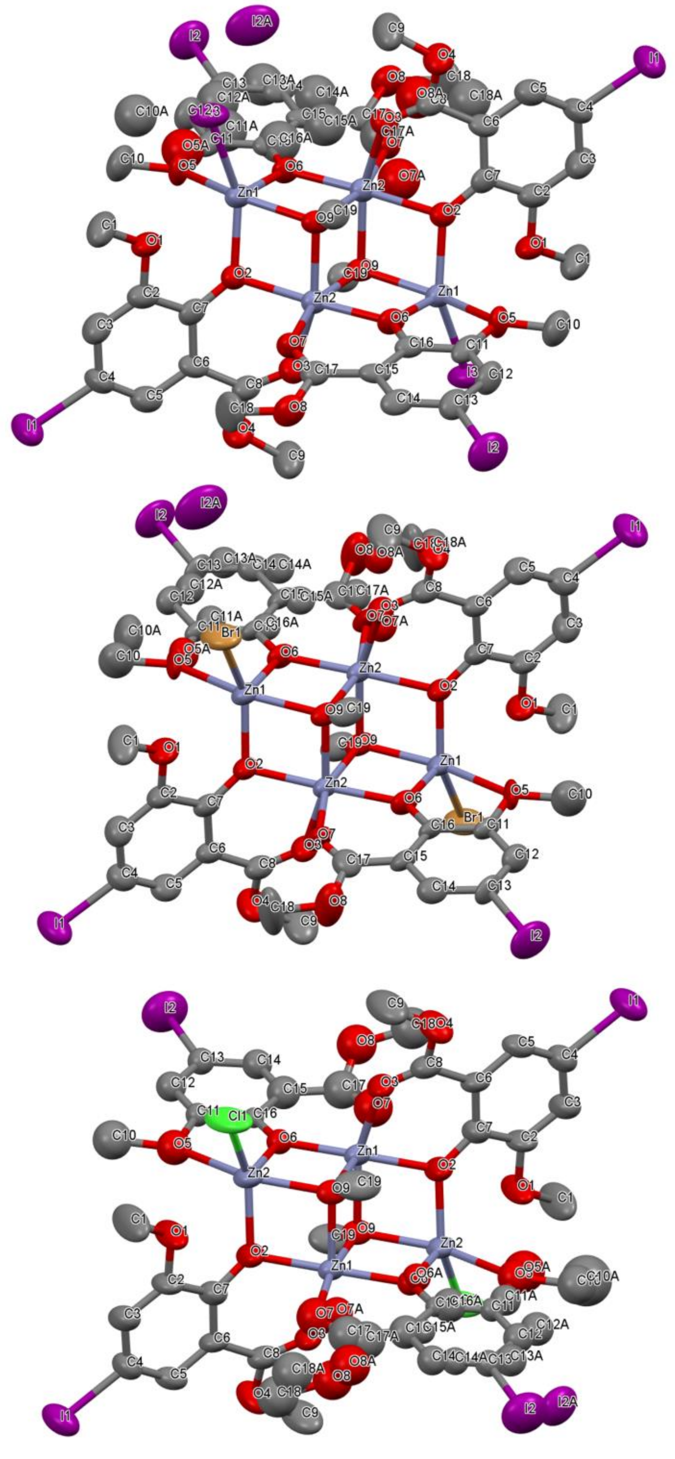

Figure S2. Crystal structure for (a) **1**, (b) **2** and (c) **3** at 173 K with thermal ellipsoids at the 50% probability level. All hydrogen atoms are omitted for clarity. Color code: grey, Zn; red, O; light grey, C; purple, I; oche, Br; green, Cl.

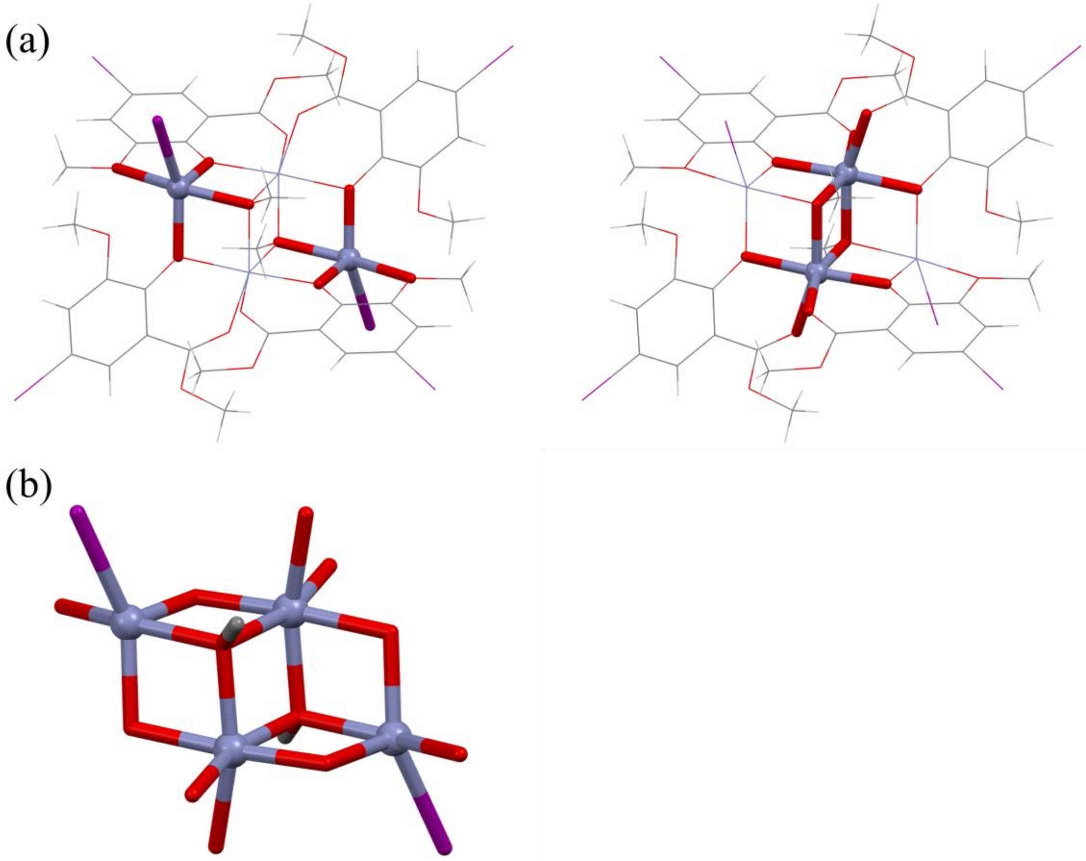

Figure S3. (a) The two kinds of Zn(II) octahedral coordination spheres in **1**. (b) Defective double-cubane core [Zn_4O_6].

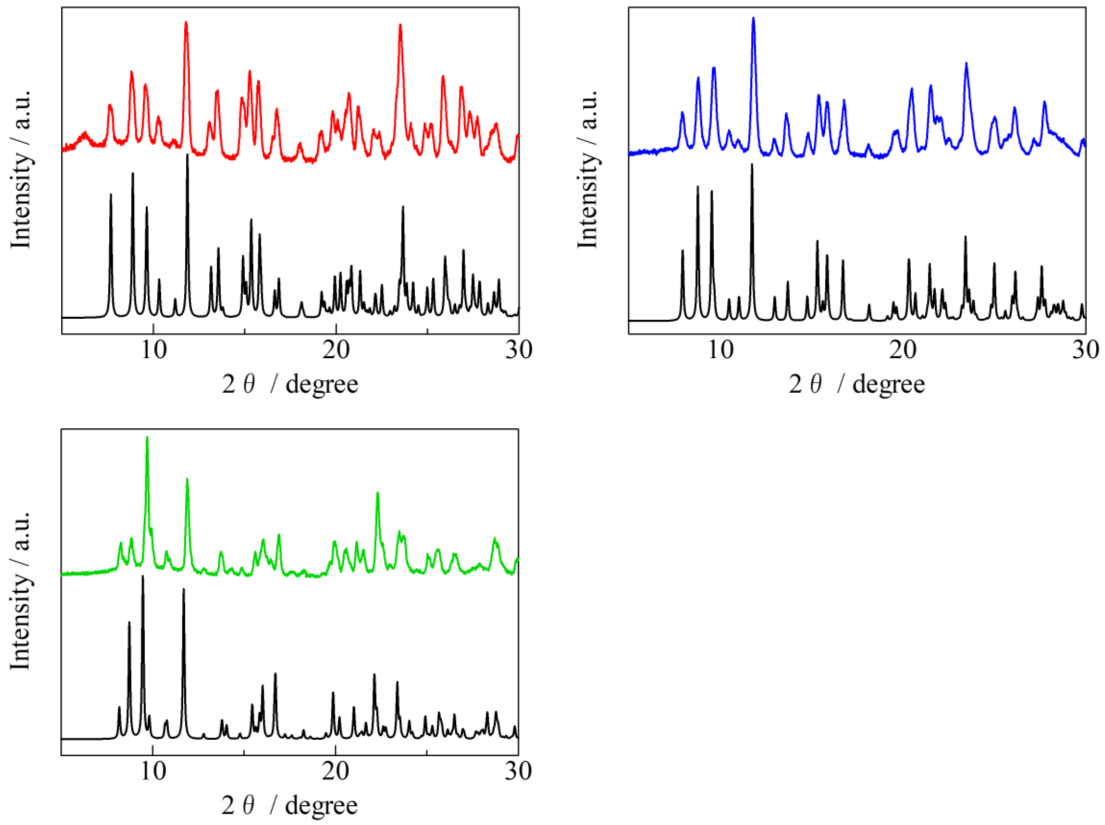

Figure S4. PXRD patterns for the microcrystals of **1** (red), **2** (blue) and **3** (green) at 298 K and their simulations (black).

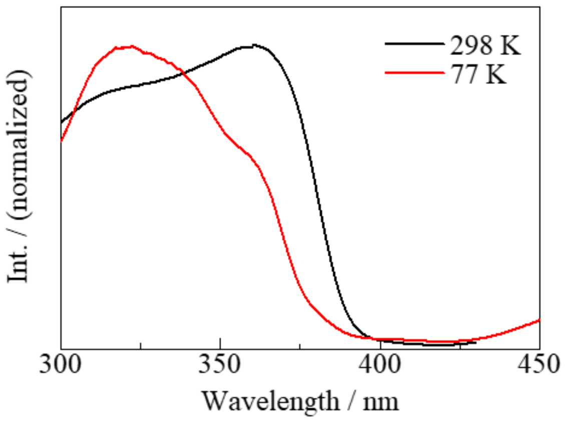

Figure S5. Excitation spectra of $\mathbf{HL^1}$ in the solid state at 298 K and 77 K.

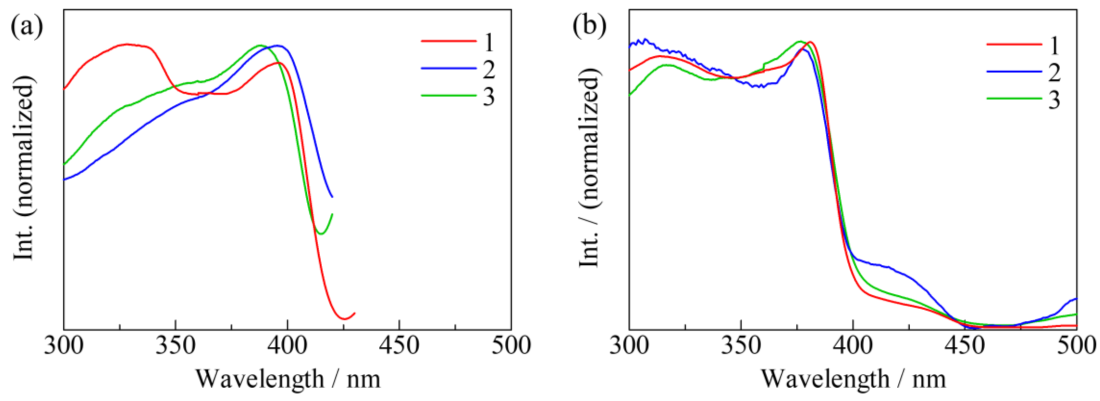

Figure S6. Excitation spectra of **1**–**3** in the solid state at (a) 298 K and (b) 77 K.

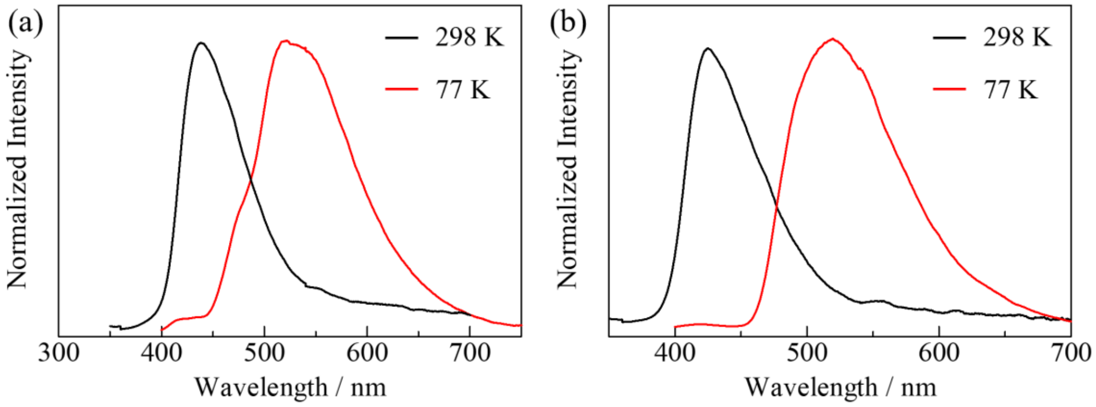

Figure S7. Emission spectra of (a) **2** and (b) **3** in the solid state at 298 K and 77 K.

Table S2. Photophysical data for **HL**[I] and **1**–**3** in the solid state at 298 K.

	λ_{em} [nm][a]	Φ[b]
**HL**[I]	473	0.02
**1**	425	0.02
**2**	438	0.02
**3**	425	0.02

[a] Emission maximum, λ_{ex} = 300 nm. [b] Emission quantum yields, λ_{ex} = 337 nm.

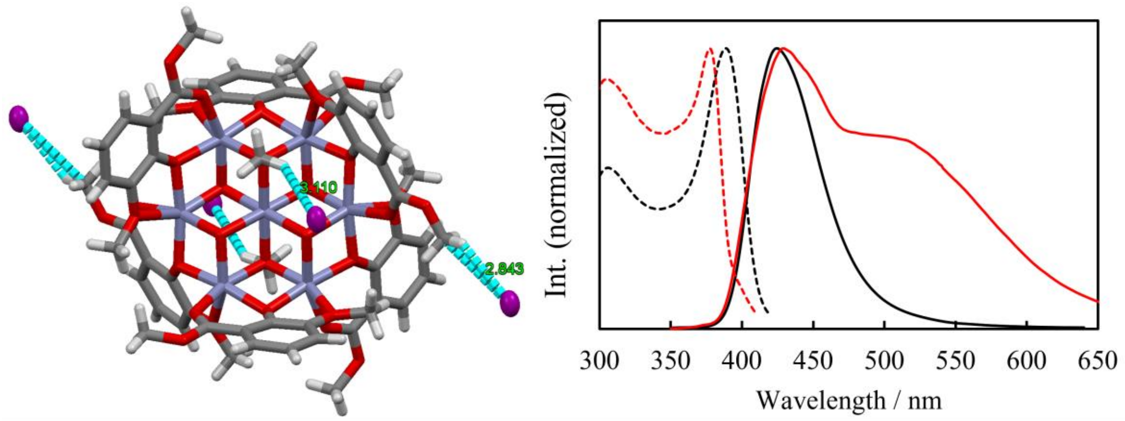

Figure S8. Emission spectrum for the heptanuclear Zn(II) cluster $[Zn_7L_6(\mu_3\text{-OMe})_2(\mu_3\text{-OH})_4]I_2$ at 298 K (black solid line) and 77 K (red solid line).[1] The emission intensity ratio of $I_{phosphorescence}/I_{fluorescence}$ at 77 K is 0.66. The dotted lines represent excitation spectra.

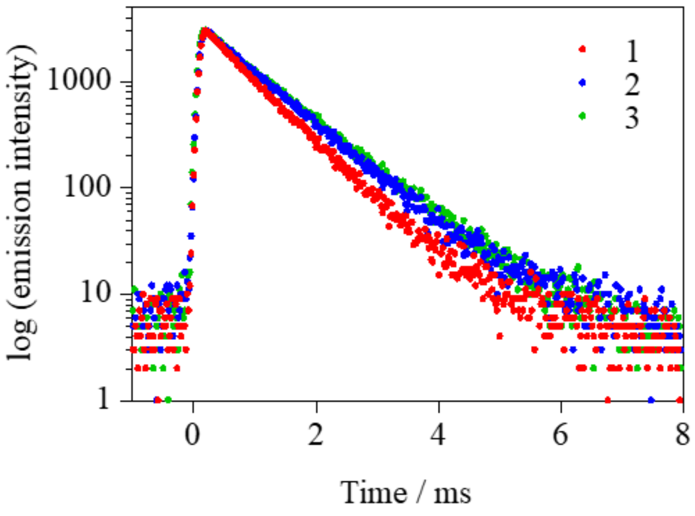

Figure S9. Emission decays of **1–3** at 77 K in the solid state (λ_{ex} = 337 nm).

Table S3. Emission lifetime parameters for **1**–**3** in the solid state at 77 K.

	τ [ms] (A) [a][b]	τ_{ave} [ms] [c]	χ^2 [d]
**1**	0.672(0.770)<br>1.03(0.230)	0.784	1.04
**2**	0.690(0.152)<br>0.943(0.848)	0.914	1.18
**3**	0.315(0.0551)<br>0.960(0.945)	0.948	0.991

[a] pre-exponential factor. [b] λ_{ex} = 337 nm. [c] Averaged emission lifetimes. [d] Fitting parameters.

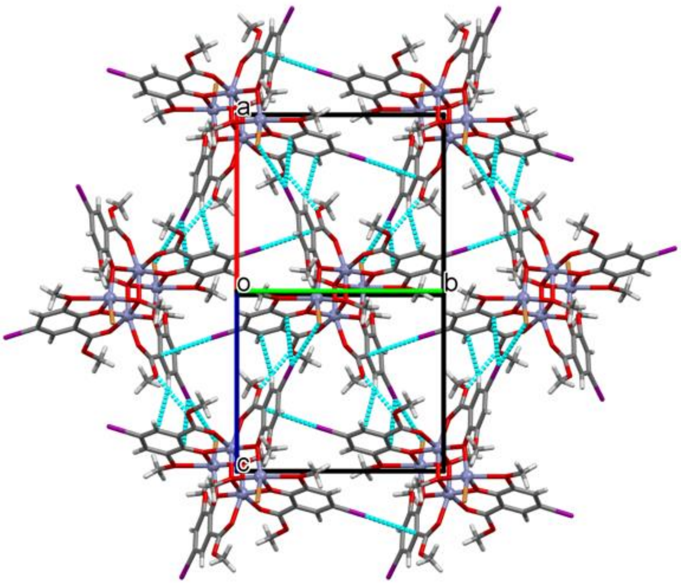

Figure S10. Crystal packing structure for **2** at 173 K. All disordered atoms are omitted for clarity. Blue-dashed lines represent halogen-related interactions (CH–I, I–Br and I–π interactions).

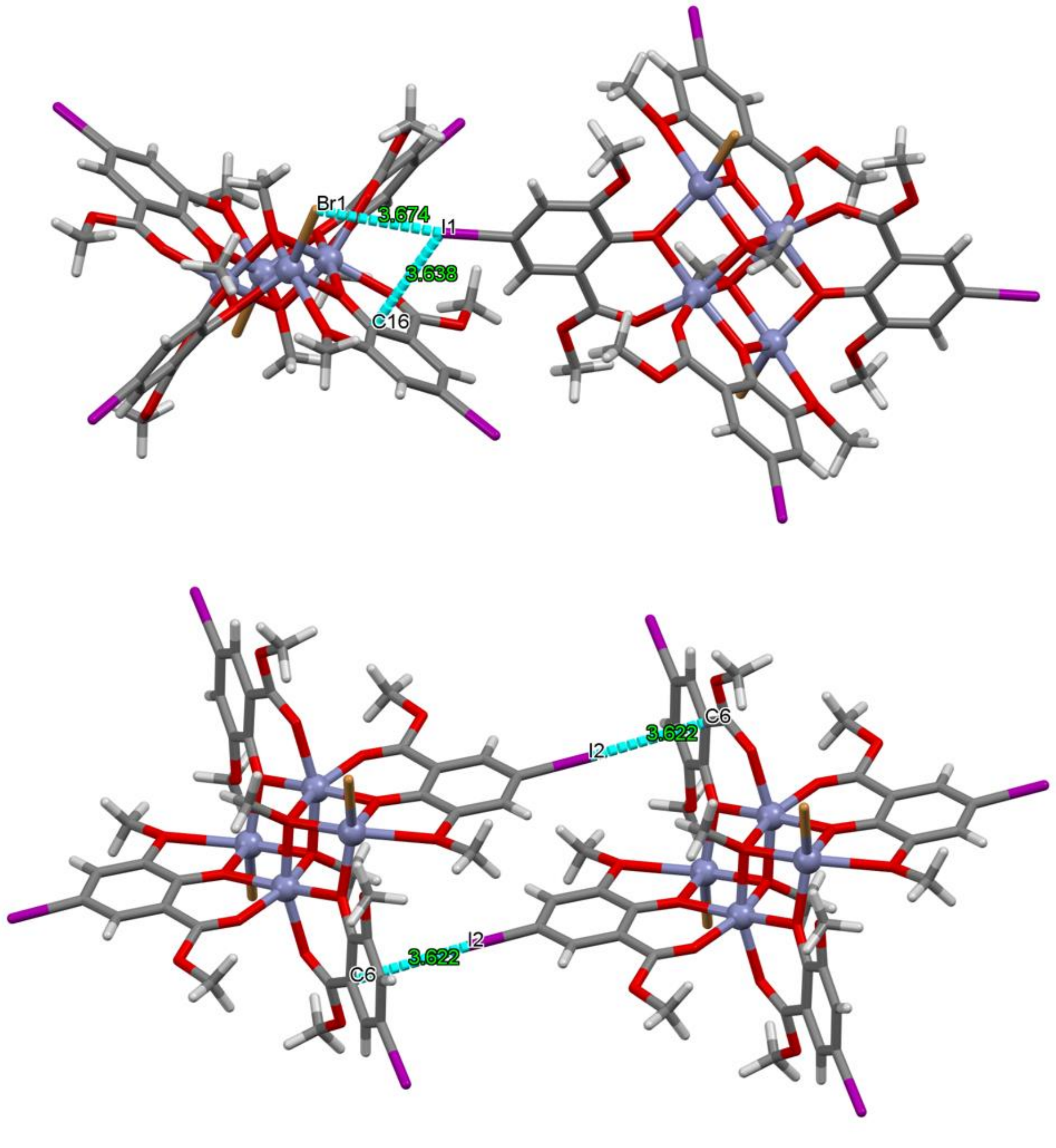

Figure S11. Intermolecular interactions of **2** at 173 K with the atom-numbering scheme. All disordered atoms are omitted for clarity.

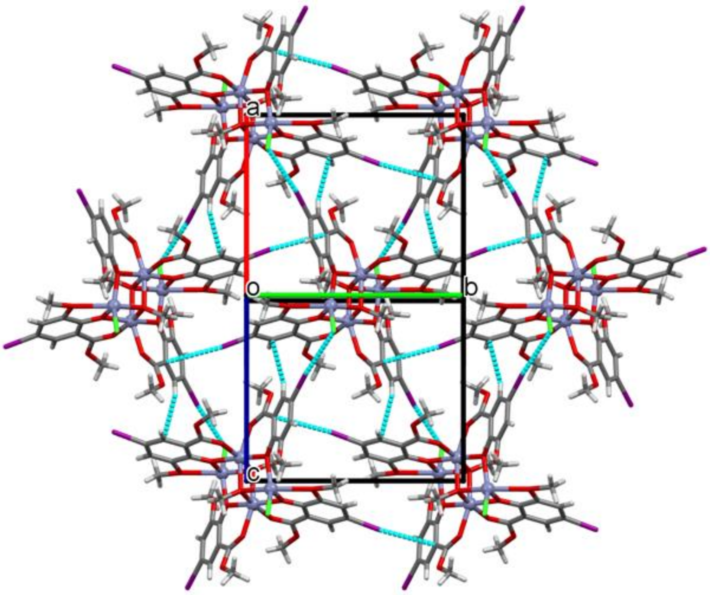

Figure S12. Crystal packing structure of **3** at 173 K. All disordered atoms are omitted for clarity. Blue-dashed lines represent halogen-related interactions (CH–I, I–Cl and I–π interactions).

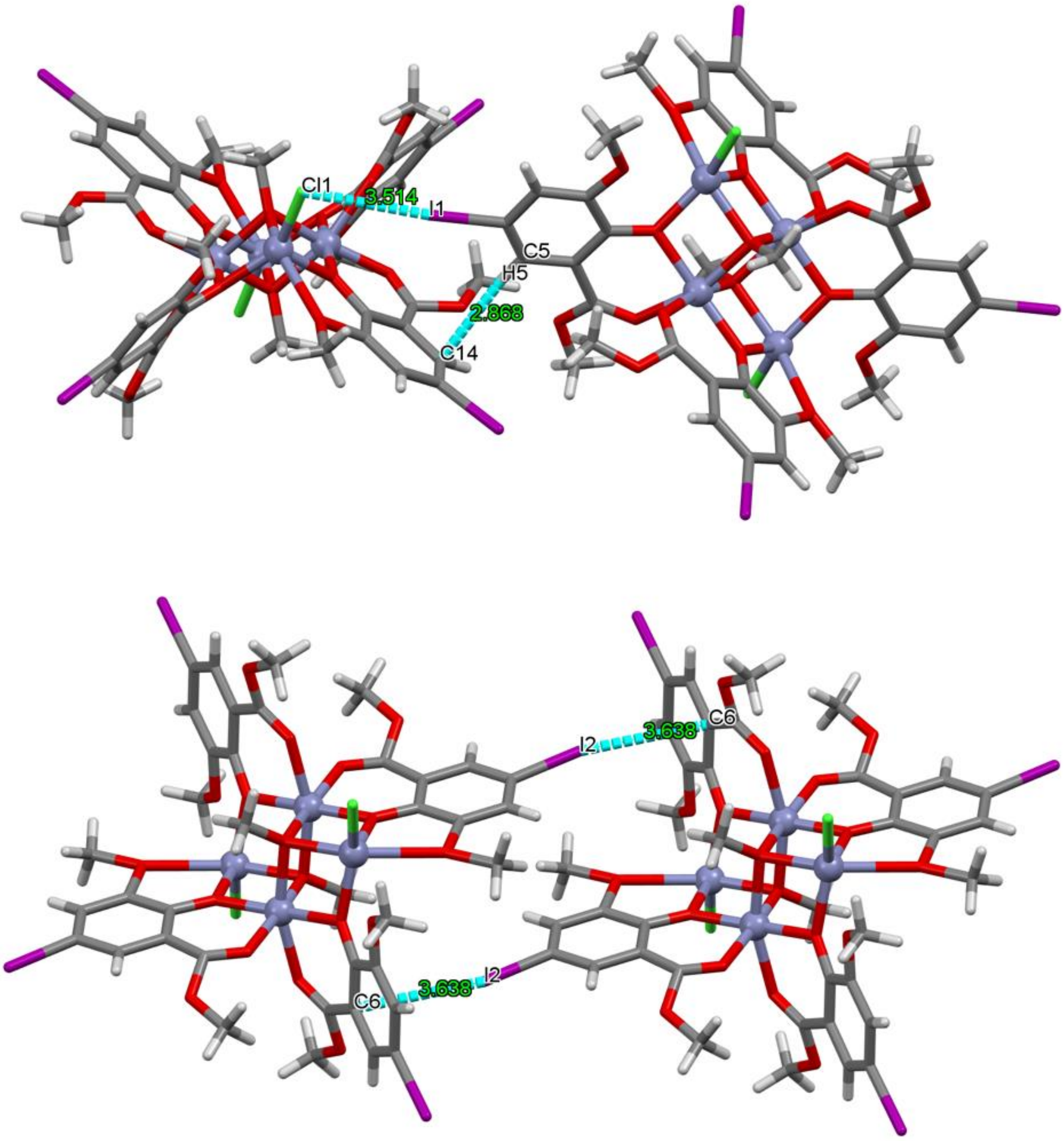

Figure S13. Intermolecular interactions of **3** at 173 K with the atom-numbering scheme. All disordered atoms are omitted for clarity.

Table S4. Selected halogen-interaction distances (Å) for **1**.

I(1)···I(3)	3.827(1)
I(1)···H(9A)–C(9)	3.142
I(1)···C(16)	3.676(7)
I(2)···C(6)	3.58(1)
I(2)···C(7)	3.657(9)

Table S5. Selected halogen-interaction distances (Å) for **2**.

I(1)···Br(1)	3.674(2)
I(1)···H(18C)–C(18)	3.0887
I(1)···C(16)	3.64(2)
I(2)···C(6)	3.622(9)

Table S6. Selected halogen-interaction distances (Å) for **3**.

I(1)···Cl(1)	3.514(6)
Cl(1)···H(9A)–C(9)	2.927
I(2)···C(6)	3.64(1)

Table S7. Crystallographic data for **HL^I**.

Compound	**HL^I**
Temperature / K	173
Formula	$C_9H_9IO_4$
Crystal system	Orthorhombic
Space group	*Pbcn* (#60)
a / Å	12.4073(4)
b / Å	9.5770(3)
c / Å	17.4191(5)
α / °	90
β / °	90
γ / °	90
V / Å^3	2069.82(11)
Z	8
D_{calc} / g cm^{-3}	1.977
μ / mm^{-1}	3.080
F(000)	1184
R_1, wR_2 ($I > 2\sigma(I)$)	0.0251, 0.0479
R_1, wR_2 (for all data)	0.0444, 0.0530
GOF	1.033
Reflections/Parameters	2111/130
CCDC	2332306

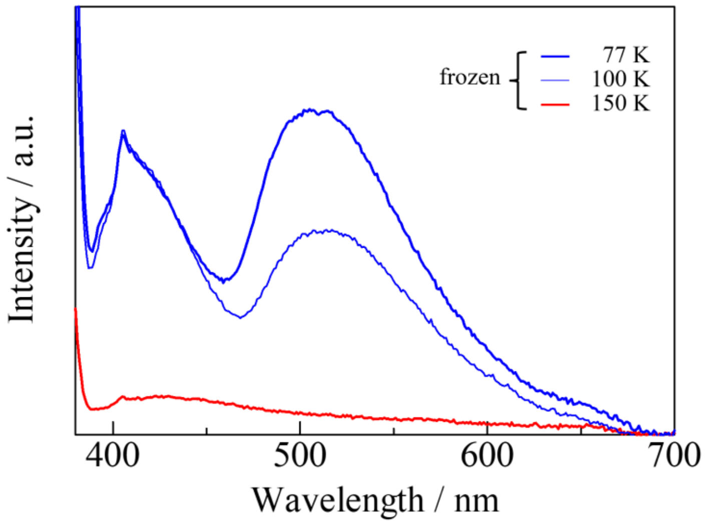

Figure S14. Temperature dependence of emission spectra for $\mathbf{HL^{I}}$ at 150–77 K in MeOH (1.0×10^{-5} M).

Reference in SI

1. F. Kobayashi, R. Ohtani, S. Teraoka, M. Yoshida, M. Kato, Y. Zhang, L. F. Lindoy, S. Hayami, M. Nakamura, *Chem. Eur. J.* **2019**, *25*, 5875−5879.
